# Biotic countermeasures that rescue *Nannochloropsis gaditana* from a *Bacillus safensis* infection

**DOI:** 10.3389/fmicb.2023.1271836

**Published:** 2023-10-18

**Authors:** Brittany Humphrey, Morgan Mackenzie, Mia Lobitz, Jenna Y. Schambach, Greyson Lasley, Stephanie Kolker, Bryce Ricken, Haley Bennett, Kelly P. Williams, Chuck R. Smallwood, Jesse Cahill

**Affiliations:** ^1^Sandia National Laboratories, Department of Molecular and Microbiology, Albuquerque, NM, United States; ^2^Sandia National Laboratories, Department of Systems Biology, Livermore, CA, United States

**Keywords:** phage therapy, crop protection, microalgae, microbiome, transplant

## Abstract

The natural assemblage of a symbiotic bacterial microbiome (bacteriome) with microalgae in marine ecosystems is now being investigated as a means to increase algal productivity for industry. When algae are grown in open pond settings, biological contamination causes an estimated 30% loss of the algal crop. Therefore, new crop protection strategies that do not disrupt the native algal bacteriome are needed to produce reliable, high-yield algal biomass. Bacteriophages offer an unexplored solution to treat bacterial pathogenicity in algal cultures because they can eliminate a single species without affecting the bacteriome. To address this, we identified a highly virulent pathogen of the microalga *Nannochloropsis gaditana*, the bacterium *Bacillus safensis*, and demonstrated rescue of the microalgae from the pathogen using phage. 16S rRNA amplicon sequencing showed that phage treatment did not alter the composition of the bacteriome. It is widely suspected that the algal bacteriome could play a protective role against bacterial pathogens. To test this, we compared the susceptibility of a bacteriome-attenuated *N. gaditana* culture challenged with *B. safensis* to a *N. gaditana* culture carrying a growth-promoting bacteriome. We showed that the loss of the bacteriome increased the susceptibility of *N. gaditana* to the pathogen. Transplanting the microalgal bacteriome to the bacteriome-attenuated culture reconstituted the protective effect of the bacteriome. Finally, the success of phage treatment was dependent on the presence of beneficial bacteriome. This study introduces two synergistic countermeasures against bacterial pathogenicity in algal cultures and a tractable model for studying interactions between microalgae, phages, pathogens, and the algae microbiome.

## Introduction

Microalgal bacterial communities (hereafter referred to as bacteriomes) coexist within the algal phycosphere in most ecosystems (Sapp et al., 2007; Seymour et al., 2017). Attempts to cultivate a bacteria-free or axenic microalgal monoculture have been shown to be unsuccessful and negatively impact growth (Droop and Elson, 1966; Jones et al., 1973) or the stability of cultures (Godwin et al., 2018). The bacteriome is selected and sustained on photosynthetic exudates produced by microalgae, and in return, bacterial community members exchange beneficial byproducts for the microalgae (Kazamia et al., 2012; Barry et al., 2016). For example, the bacteriome can stimulate the growth of specific microalgae by producing growth phytohormones or supplying limiting nutrients, such as a B-vitamin or fixed nitrogen (Mönnich et al., 2020; Mars Brisbin et al., 2022). Conversely, phycosphere bacteria manipulate microalgae by secreting algicidal molecules that selectively kill certain species or by competing for essential nutrients. However, the stability, specificity, and currencies of chemical exchange between microalgae and the established bacteriome require further characterization (Liu et al., 2020; Schambach et al., 2020).

Marine microalgae are a diverse group of photosynthetic microorganisms capable of producing high-value renewable products from minimal inputs such as seawater, micronutrients, light, and CO_2_ (Sheehan et al., 1998; Dismukes et al., 2008; Schenk et al., 2008; Davis et al., 2011; Gimpel et al., 2013)_._ Algal biotechnology relies heavily on bioprospecting for strains with high biomass and lipid productivities and/or genetic manipulation to maintain and/or enhance these traits in industrial settings. Despite advancements with these strategies, production target goals have still not been met in large part due to inconsistencies in production pipelines (Krishnan et al., 2021; Wiatrowski and Davis, 2021). For example, raceway pond systems are cost-effective for large-scale microalgal production; however, these open systems are prone to pathogens and pests. As much as 30% of crops are lost to biological contamination (McBride et al., 2014). Despite their longstanding pervasiveness, the ways in which bacterial pathogens cause algal pond collapse are not well understood (Brussaard, 2004; Cheng et al., 2004; Pei et al., 2007). Current methods to control bacterial infections in large-scale microalgal systems are limited to non-specific treatment strategies. Many of them, like antibiotics, are toxic to the environment and lead to drug resistance. Consequently, the use of antibiotics to treat pond crashes will alter or destroy the bacteriome, resulting in reduced microalgal growth (Liu et al., 2020; Schambach et al., 2020) and will likely leave cultures more susceptible to other harmful pathogens or pests (Fisher et al., 2019).

*Nannochloropsis* spp. are eukaryotic marine microalgae that have emerged as a leading microalgal platform for the production of high-value petroleum replacement products and nutraceuticals such as omega-3 fatty acids (Liu et al., 2017; Zanella and Vianello, 2020). Previous research showed that the bacteriome of *Nannochloropsis* spp. promotes algal growth (Liu et al., 2020; Schambach et al., 2020) and is protective against rotifer infection (Fisher et al., 2019). A recent study identified a common soil bacterium, *Bacillus pumilus*, that inhibits the growth of *Nannochloropsis* spp. (Fulbright et al., 2016). *Bacillus* spp. are not known to be part of the native *N. gaditana* microbiome (Schambach et al., 2020). However, *Bacillus* spp. show lytic activity against multiple microalgae (Sheehan et al., 1998; McBride et al., 2014; Mars Brisbin et al., 2022). The lack of countermeasures available for addressing bacterial pathogens in microalgae motivated us to explore the use of phages in microalgal systems. Unlike antibiotics, phages are capable of species-specific targeting (de Jonge et al., 2019); thus, phage treatment would be expected to precisely reduce or eliminate the targeted pathogen without affecting the bacteriome, leaving its growth-promoting function intact. As a proof-of-concept, we tested whether *N. gaditana* can be rescued from a *Bacillus* infection (Fulbright et al., 2016) in cultures seeded with phage Leo2 (Badran et al., 2018) while monitoring the dynamics of the bacteriome during phage rescue. We then tested if the bacteriome itself is protective against *Bacillus* infection and whether the phage and algal bacteriome work together to ward off *Bacillus* pathogenicity to microalgae.

## Materials and methods

### Microalgae, bacteria, and phages used in this study

*N. gaditana* CCMP526 (*N. gaditana* hereafter) was obtained from Dr. Matthew Posewitz at the Colorado School of Mines in Golden, Colorado. *Bacillus safensis* was originally identified as *Bacillus pumilus* by 16S rRNA sequencing (Badran et al., 2018). Herein, whole-genome sequencing showed that this isolate was *B. safensis. B. safensis* and *Bacillus* phage Leo2 were kindly provided by Badran et al. (2018).

### Algal cultivation

*N. gaditana* stock cultures were maintained photoautotrophically in a Percival incubator (Percival Scientific, United States) at 25°C, under 215 μmol m^−2^ s^−1^ warm white LED light at 4000K color with a 12-h diurnal cycle. Stock cultures were continuously mixed on an orbital shaker at 150 rpm. Initial cultures were seeded into 4-ml F2N•NO_3_ media from F2N•NO_3_ plates. The F2N•NO_3_ media used for subculturing (supplementation of marine broth described below) was F2N•NO3 and consisted of the following components: 34.12 g/L Crystal Sea Oceanic Sea Salt mix, 10 mM Tris HCl, 724 μM (100 mg/L) NaH_2_PO_4_•H_2_O, 25 mM (2.16 g/L) NaNO_3_, 0.0045 μM (0.76 mg/L) MnSO_4_•H_2_O, 0.00015 μM (0.0365 mg/L), Na_2_MoO_4_•2H_2_O, 0.0004 μM (0.115 mg/L) ZnSO_2_•7 H_2_O, 0.00025 μM (0.07 mg/L) CoSO_4_•7H_2_O, 0.0002 μM (0.034 mg/L) CuCl_2_ •2H_2_O, 0.0585 μM (0.023 g/L), Fe(NH_4_)_2_(SO_4_)_2_•6H_2_O, 0.0585 μM (0.022 g/L) Na2EDTA•H_2_O, 1.899 μM (675 μg/L) Cobalamin (B12), 0.51 μM (125 μg/L) Biotin, 8.15 μM (2.75 mg/L) Thiamine HCl. Vitamin stocks were prepared in 50-mM HEPES buffer. F2N•NO_3_ was filter-sterilized with a 0.2-micron Nalgene Rapid-Flow Sterile Vacuum Filter (Thermo Fisher Scientific, United States). Stock cultures were routinely passaged in F2N•NO_3_ liquid media during mid-log phase growth.

### Preparation of *N. gaditana* for exposure to bacteria and phages

*N. gaditana* was cultivated in 50-ml F2N•NO_3_ until mid-log phase (~10^7^ cells/mL) in 250-ml baffled flasks before experiments. For the subsequent experiments, 50 ml of subcultures were centrifuged at 7,000 × *g* for 10 min and resuspended in 20 ml of marine broth-supplemented media to achieve a cell density of ~5 × 10^6^ cells/mL in 125-ml baffled flasks. The marine broth-supplemented media consisted of 1:4 v:v Difco Marine Broth 2216 (BD Diagnostics) and F2N•NO_3_ (MB/F2N•NO_3_).

### Preparation of *Bacillus* for exposure to microalgal cultures

A single colony of *Bacillus safensis* was inoculated in 4 ml of Luria Broth (LB) and grown overnight at 30°C on an orbital shaker at 200 rpm. The next day, *B. safensis* was subcultured 1:100 in 50-ml LB media in a baffled flask and incubated at 30°C with aeration to an OD of 1.3 (~8 × 10^8^ CFU/ml). The subculture was transferred to a 50-ml conical tube and centrifuged at 8,000 × *g* for 7 min at room temperature. The pellet was washed twice in 1:4 v/v MB/F2N•NO_3_ before diluting to the desired multiplicity of infection (MOI = cell ratio of *Bacillus:Nannochloropsis*). In total, 100 μl of diluted cells or 100 μl of 1:4 v/v MB/F2N•NO_3_ media were introduced into 20 ml of cultures seeded with 5 × 10^6^ *N. gaditana* cells.

### Phage stock maintenance and preparation of Leo2 for exposure to microalgal cultures

Leo2 lysates were produced by mixing ~1 × 10^6^ plaque-forming units (PFU) with 100 μl of *B. safensis* culture, inoculated into molten LB agar (0.6% agar, supplemented with 5 mM CaCl_2_ and 10 mM MgCl_2_), and plated onto LB agar plates. After overnight incubation, 5 ml of SM buffer (Teknova, United States) was added to each plate and agitated by a rotary shaker at room temperature for ~1 h. The SM/top agar mixture was scraped into 50-ml conical tubes, vortexed for 10–15 s, and then centrifuged for 30 min at 5,000 × g, at 4°C. The supernatant was filter sterilized and then titered as described below. To prepare Leo2 for phage treatment experiments, 5–10 ml was dialyzed twice in 2 L of 34 g/L Crystal Sea (Marine Enterprises International) using Spectra pre-wetted RC membranes (Repligen part # 132544). After dialysis, the Leo2 suspension was filter sterilized, and 65 μl of phage or sterile crystal sea (control) was added to cultures at a final concentration of ~5 × 10^7^ PFU/ml.

### Bacteriome-attenuated *N. gaditana* culture

The bacteriome-intact and bacteriome-attenuated cultures were propagated from a parent culture of *N. gaditana* that was passaged once on an antibiotic plate containing 100 μg/ml ampicillin, 30 μg/ml chloramphenicol, 100 μg/ml streptomycin, and 100 μg/ml tetracycline (Schambach et al., 2020). Two daughter cultures were propagated separately after this treatment, 1 year prior to the start of the current study. During that time, one culture (denoted *Ng*^*MB*+^) spontaneously recruited a new microbiome. For each experiment, we confirmed that *Ng*^*MB*−^ was bacteriome-attenuated by 16S rRNA amplicon sequencing. We also confirmed that *Ng*^*MB*−^ did not produce bacterial colonies when 10 μl of samples were spread on MB and LB plates, even after >1-week incubation.

### *N. gaditana* bacteriome transplant using fluorescence-activated cell sorting

The BD FACSAria Fusion cell sorting flow cytometer (BD Biosciences, San Jose, CA) was used to sort bacteriome members from the microalgae cells using a 70-μm nozzle at 70 psi. In addition, the 1.0 neutral density filter was inserted into the beam path to allow simultaneous visualization of the whole microalgae and bacteria populations. Subpopulations of the microalgae and bacterial cells were defined by their relative size and microalgal chlorophyll fluorescence in the PE-Cy5 (Ex: 561 nm, Em: 710/50) channel. *N. gaditana* cultures grown to ~5 × 10^7^ cells/ml were then sorted into two tubes with a flow rate of ~7,000 cells/min until 5 ml of culture was sorted. Microalgae cells with chlorophyll and aggregate bacterial cells without chlorophyll were collected into two separate tubes. After sorting, separate microalgae and bacterial cells were gently centrifuged (5,000 × g) for 5 min, washed, and resuspended in F2N•NO_3_ twice before final resuspension (below). The transplanted bacteriome cultures were then checked for the correct size and chlorophyll content before mixing with pelleted microbiome-attenuated microalgae from a 5-ml culture to match a similar ratio to the parent culture. The transplanted bacteriome–microalgae mixture was resuspended in 5 ml of F2N•NO_3_ media and grown at 25°C under 215 μmol m^−2^ s^−1^ warm white LED light at 4000 K color with a 12-h diurnal cycle. Subsequently, 10 μl of the cell-sorted bacterial fraction were plated on F_2_N•NO_3_ and then grown for 21 days under the conditions described above. No microalgal growth was observed in this medium indicating successful sorting of microalgae from bacteria.

### Sampling and measuring *N. gaditana* cell density

Samples were collected on the days indicated for each experiment. 240 μl aliquots were taken from each flask, with half the volume used to titer bacteria and the remainder used to titer phages (described below). An additional 50 μl of culture was taken from each flask for daily cell counts, and diluted as needed using media. On the indicated days, 667 μl of culture was taken from each flask, and replicates were pooled for DNA extraction and 16S sequencing.

Daily cell counting employed the BD FACSAria Fusion cell sorter using a 70 μm nozzle and a 1.0 neutral density filter. Samples were run for 15 s each, total events were collected, and the microalgae population was gated using the chlorophyll autofluorescence signal detected by the PE-Cy5 channel. To convert between total events and approximate cell density in cells/ml, semi-daily flow rates were measured using water and calculated in ml using the following equation: Cytometer flow rate = (W_i_ – W_f_) – 0.0287, where W_i_ is the initial weight of 1 ml of water, W_f_ is the final weight of the water after 5 min of uptake by the cell sorter, and 0.0287 is correcting for the initial uptake of sample inherent to the system. On average, the flow rate was 0.27 μL/s. The microalgal cell density could then be calculated using the following equation: Cell density (cells/ml) = (GE)/(CFR ^*^ t), where GE is the number of gated events, CFR is the cytometer flow rate in ml/s, and t is the time in seconds the samples were run.

### Titering phages and *Bacillus*

Phage-containing samples were centrifuged for 2 min at 18,000 × *g* to separate phages from live cells. Bacterial lawns were formed by mixing 100 μl of *B. safensis* with 4 ml of 0.8–1.0% molten top agar and immediately plating on an LB plate. Phage titering was performed using the standard double-layer agar method. Typically, 100 μl of phage suspensions was serially diluted in 900 μl of SM buffer, and 10 μl of the dilutions was spotted on the lawn in triplicate. After overnight incubation at 30°C, single plaques from the dilution containing the highest number of countable plaques were enumerated. The titer of *B. safensis* in microalgae cultures was determined by viable plate counts on LB. We found that after one day of incubation, LB was selective for *B. safensis*, i.e., the plating conditions did not support the growth of the microalgal bacteriome members. 100 μl of samples was withdrawn from microalgae cultures and diluted in 900 μl LB in serial increments, and 10 μl droplets from each dilution was spotted on LB plates in triplicates. After overnight incubation at 30°C, single colonies from the dilution containing the highest number of countable colonies were enumerated.

### Microalgal bacteriome 16S sequencing

#### DNA extraction and quantification

Microalgal microbiome samples collected for 16S sequencing were processed using the MP Biomedical FastDNA Spin Kit for Soil (SKU 116560200-CF). Extracted gDNA was stored at −20°C until processing and outsourcing for 16S sequencing. Samples collected for 16S rRNA gene sequencing were quantitated with either a Qubit 3.0 Fluorometer (Life Sciences) using the High Sensitivity (HS) DNA assay (Catalog Number Q33231) or a NanoDrop One Fluorospectrometer (ThermoFisher) to quantify the concentration of genomic DNA.

#### Sequencing

Sequencing was conducted by Zymo Research Corporation and their ZymoBIOMIC 16S sequencing service. The ZymoBIOMIC 16S rRNA gene primers (forward CCTACGGGGNGGCWGCAG, reverse GACTACHVGGGTATCTAATCC) were used to amplify the V3–V4 regions of the 16S rRNA gene. The targeted library preparation, sequencing, and bioinformatics analysis were performed as described previously (Whitehead et al., 2022). We have reported the normalized relative abundance of each distinct operational taxonomic unit (OTU) with and without chloroplast sequences, as indicated in the results.

### *B. safensis* whole-genome sequencing

*B. safensis* gDNA was purified using the MP Biomedical FastDNA Spin Kit (described above) according to the manufacturer's instructions, and 500 ng of sample was sequenced by Azenta Life Sciences (MA, United States) using the Illumina MiSeq platform (2 × 250 bp). After demultiplexing, paired-end read files (nominally for 250 bp at each end) had 667,491 entries for read 1 and 848,505 entries for read 2. Their assembly was performed using SPAdes v3.9.0 in default mode. Scaffolds below 500 bp were rejected, and a large gap was noted among the remaining scaffolds for coverage values; the 13 scaffolds with coverage of 53 or higher were retained, while the remaining 92 had coverage less than 2, were shorter than 1,300 bp, and were rejected. The final 13-scaffold assembly totaled 3.677 kbp with an N50 of 864564.

## Results

### *Bacillus safensis*: a highly virulent pathogen of *N. gaditana*

**Figure 1:**
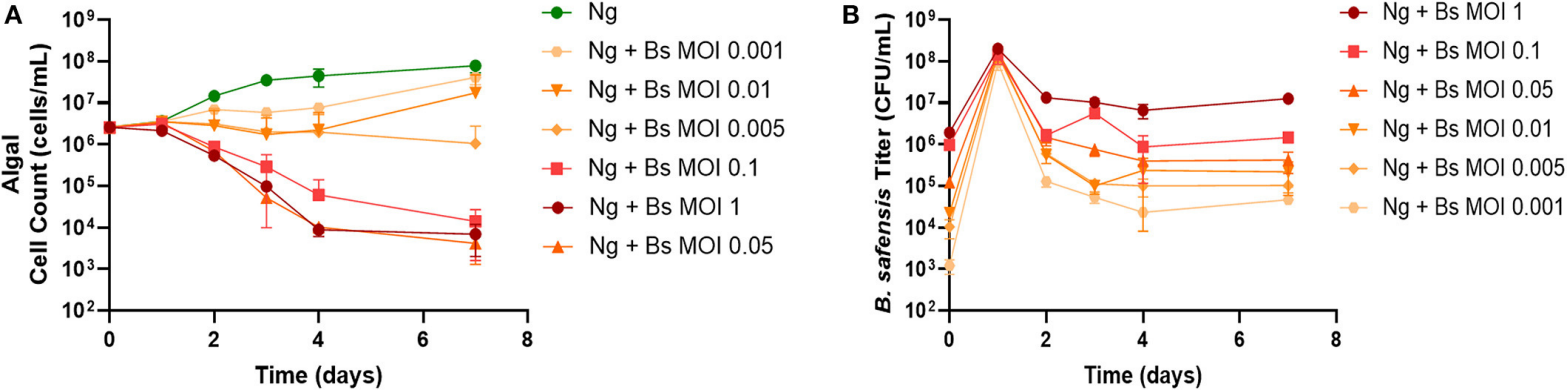
Pathogenicity of *B. safensis. B. safensis*. (Bs; originally thought to be *B. pumilus*) was added to cultures at time = 0 at the indicated MOIs (ratio of bacteria: algae) to discern the minimum inhibitory concentration for *N. gaditana* (Ng) growth. **(A)** Growth profile obtained from *N. gaditana* cell counts using flow cytometry. **(B)** Profile of *B. safensis* titer over time. *B. safensis* titers were obtained by serial dilution of samples from each co-culture flask. The values plotted represent the average and standard deviation, represented by error bars, of three independent cultures.

To test whether phages can be used to eliminate pathogens from microalgal bacteriomes, we chose *N. gaditana* as a model system based on a recent study showing that *B. pumilus* inhibits the growth of *Nannochloropsis* spp. (Fulbright et al., 2016). We could not obtain the *B. pumilus* isolate used in that report and therefore chose to work with a different *Bacillus* isolate. We began by characterizing its virulence to *N. gaditana*. Surprisingly, every multiplicity of infection (MOI = cell ratio of *Bacillus* to *N. gaditana*) tested showed reduced algal cell count over time (Figure 1A) (MOI: 0.001, 0.005, 0h.01) or caused a sharp decrease, or “crash,” in the *N. gaditana* cell count over time (MOI: 0.05, 0.1, 1). Figure 1B shows that when *Bacillus-*infected cultures were seeded at the reported multiplicities, the titer of *Bacillus* in cultures reached ~1 × 10^8^ CFU/ml by day 1. Notably, the titer abruptly declined ~1–3 log on day 2, with reductions proportionate to the starting inoculum, i.e., the lowest MOI showed the greatest decrease (Figure 1B). After this period, the titer of *Bacillus* in each culture remained relatively stable until the end of the experiment.

To understand why our *Bacillus* isolate showed pathogenicity at ~4 log lower doses than previously reported, we performed whole-genome sequencing and assembly to investigate strain or species-level differences. The genome was compared to all 65,703 species representative genomes from GTDB release 207 (Parks et al., 2022) using our script Speciate (Mageeney et al., 2023). Average nucleotide identity (ANI) values above 90% were obtained for only six of the species representatives, all from the genus *Bacillus*. The highest ANI (98.91%, with an alignment fraction of 95.84%) was found for *B. safensis*, well within the cutoff of 95% ANI that GTDB had set for each of these candidate species. The next highest ANI (93.16% for *B. australimaris*) was well below this cutoff, and the remaining candidates (including *B. pumilis* and *B. pumilis_N*) had ANI values under 92%. These data unambiguously indicated that this isolate was *Bacillus safensis. B. safensis* has not been reported to be pathogenic to microalgae but is closely related to *B. pumilus* (Branquinho et al., 2014).

### Phage rescue of *N. gaditana* cultures infected with *Bacillus safensis*

**Figure 2:**
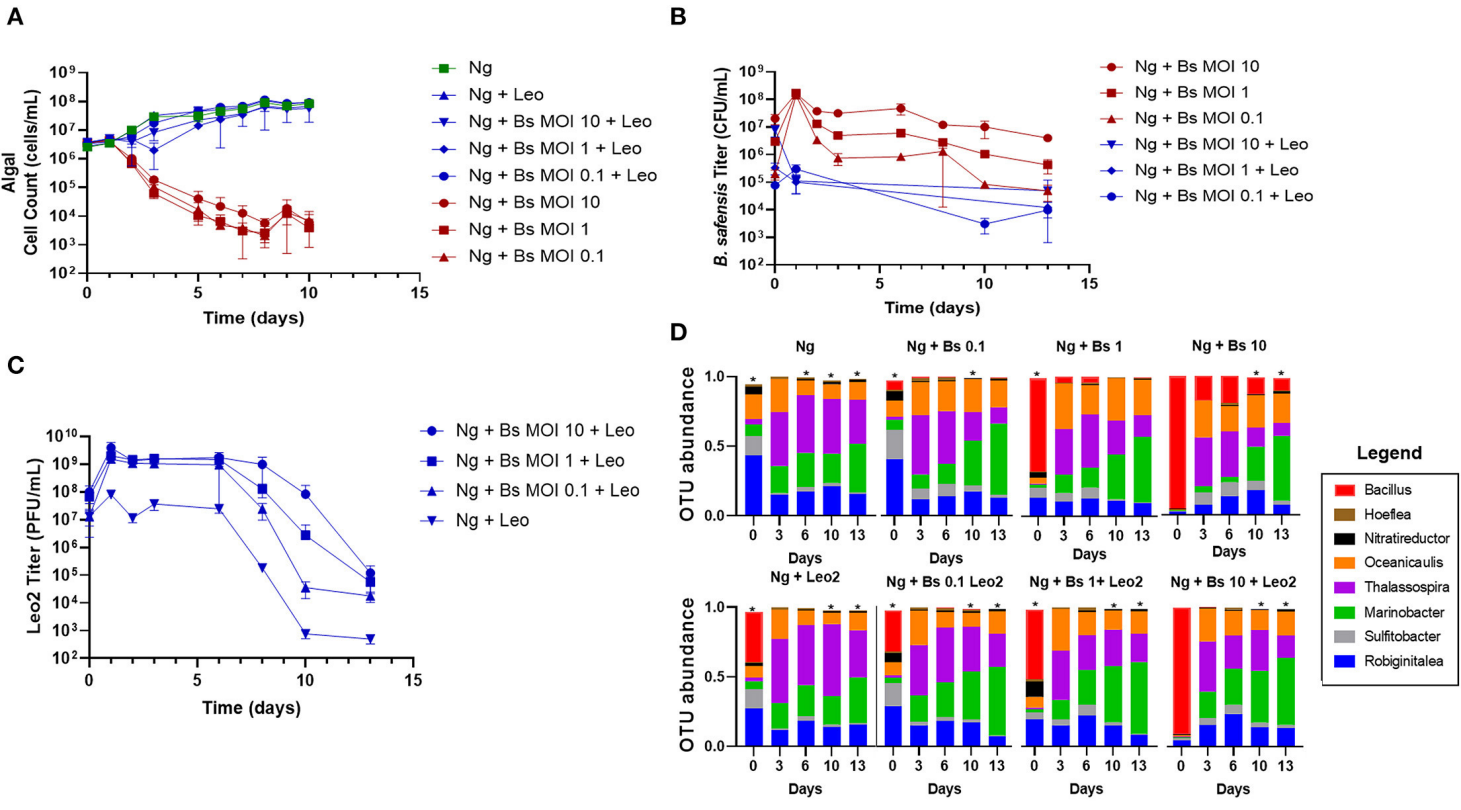
Phage rescue of *N. gaditana* cultures infected by *B. safensis*. Cultures containing *N. gaditana* (Ng) and/or *B. safensis* (Bs) and/or Leo2 (Leo) were co-cultured at *time* = 0 at the indicated MOIs (ratio of bacteria: algae) for Bs. For phage-seeded cultures, the starting concentration was ~5 × 10^8^ PFU/ml. **(A)** Growth profile obtained from *N. gaditana* cell counts using flow cytometry. **(B)** Profile of *B. safensis* titer over time. *B. safensis* titers were obtained by serial dilution of samples from each co-culture flask. **(C)** The titer of free phages in cultures containing Ng and/or Bs. The values plotted represent the average and standard deviation, represented by error bars, of three independent cultures. **(D)** Relative OTU (operational taxonomic unit) abundance for bacterial 16S rRNA sequences. Asterisks within this panel indicate when the eight core bacteriome members listed in the figure key do not solely compose the entirety of the OTU data for a sample on a particular day.

The above findings identified a range of starting concentrations of *B. safensis* that were pathogenic to *N. gaditana*. Three multiplicities (MOI 0.1, 1, and 10) were selected to test whether cultures could be rescued by phage Leo2 (Badran et al., 2018). Each *N. gaditana* culture that was inoculated with both *B. safensis* and Leo2 showed comparable growth to the untreated control microalgae, whereas the *Bacillus*-infected cultures lacking phage began to crash within the first 2 days (Figure 2A). We also monitored the Leo2 titer in the supernatant throughout the experiment and found that it was proportionate to the starting inoculum of *B. safensis* (Figures 2B, C). As with *B. safensis*, the titer of Leo2 in cultures was highest on day 1 (Figure 2C); however, it was relatively stable for 6 days, after which there were 1–4 log decreases in the titer by day 10. As observed in Figure 1, the titer of *B. safensis* in *N. gaditana* cultures lacking phage increased to ~1 × 10^8^ CFU/ml on day 1 and decreased afterward (Figure 2B). Cultures treated with Leo2 showed a 3-log reduction in the *B. safensis* titer on day 1. Taken together, these data show that phage can rescue *N. gaditana* cultures from a *B. safensis* infection.

Given the importance of the bacteriome for microalgae growth (Liu et al., 2020; Schambach et al., 2020), we next investigated whether the phage rescue altered the balance of the bacteriome relative to controls lacking phage or *B. safensis*. We extracted DNA from each group of cultures at days 0, 3, 6, 10, and 13 and used 16S rRNA sequencing to monitor the relative abundance of members over time. 18 operational taxonomic units (OTUs) of the bacteriome were identified; 14 were resolved to the genus level, 3 were identified at the family level (Phycisphaeraceae, Rhodobiaceae, and Rhodobacteraceae), and 1 OTU from Gammaproteobacteria was not identified beyond its class. By OTU counts, the most abundant genera, or “core” members, were *Thalassospira, Marinobacter, Oceanicaulis, Robiginitalea, Nitratireductor, Hoeflea*, and *Sulfitobacter*. The less abundant genera were *Limnobacter* and *Methylophaga*. *B. safensis*-infected cultures showed a reduced OTU ratio for *Thalassospira* and *Nitratireductor* and an increased ratio for *Oceanicaulis* relative to the *N. gaditana* control (Figure 2D, Supplementary Figure 1, Supplementary Table 1).

The OTU ratios for the lowest MOI culture (0.1) treated with phage were similar to the *N. gaditana* control until day 13, when a higher ratio of *Thalassospira* was observed relative to the *Bacillus*-treated, phage-free culture. However, the residual DNA from Leo2's host present in the phage lysates contributes to the apparently increased *Bacillus* ratio since it is also detected in the *Bacillus*-free Leo2-seeded control (Figure 2D, Ng +Leo2 set). Since *N. gaditana* is reported to have one chloroplast per cell (Hamidi et al., 2014), we used chloroplast 16S sequences to gauge the relative abundance of the bacteriome vs. the microalgae (Supplementary Figure 1), which shows high similarity between phage-rescued and control cultures.

### The role of the algal bacteriome for pathogen resilience

**Figure 3:**
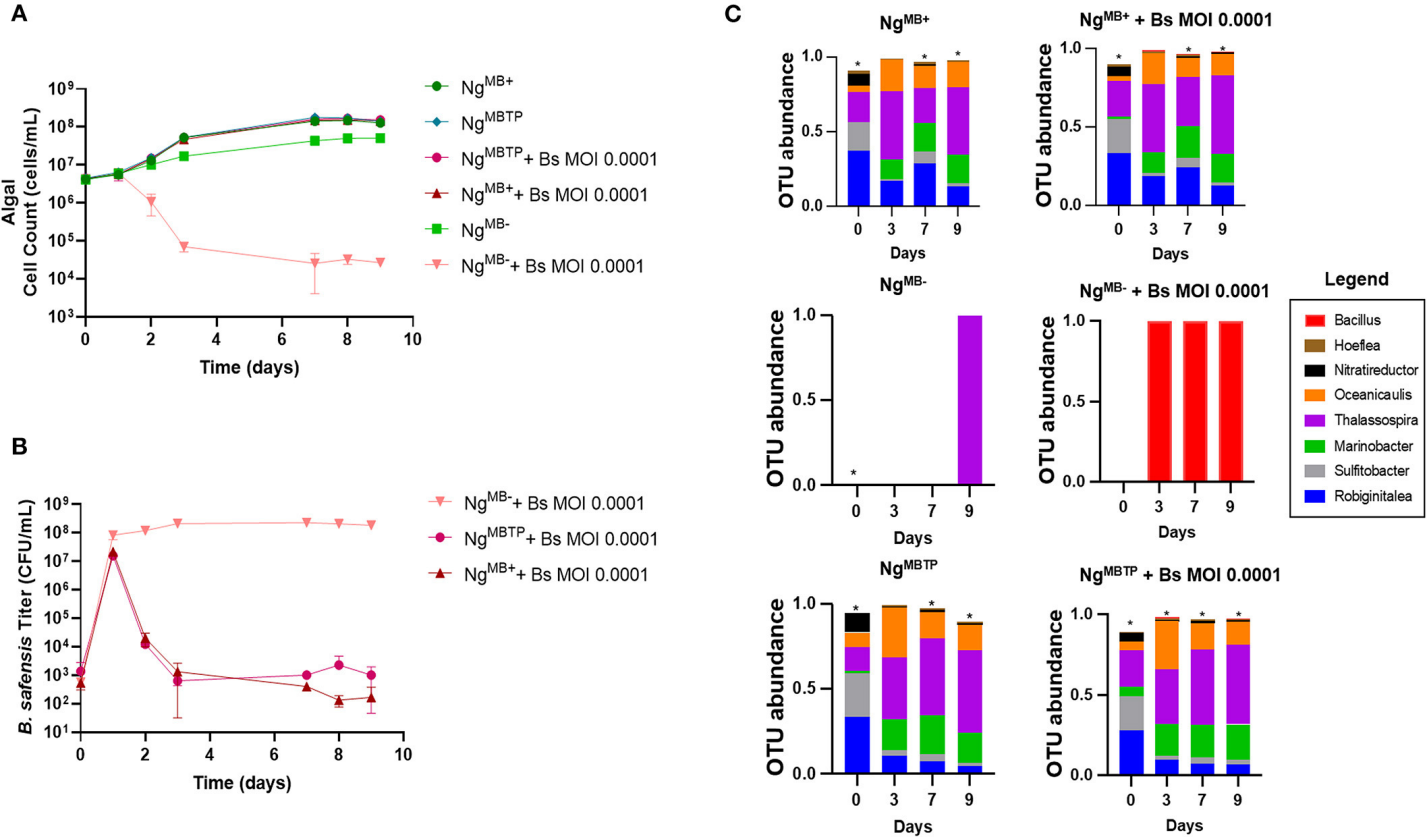
The protective role of the microbiome. Our first demonstration of the protective role of the microbiome utilized three distinct *N. gaditana* (Ng) cultures that were infected with *B. safensis* (Bs) at the indicated MOI (ratio of bacteria: algae). *Ng*^*MB*+^ = a control *N. gaditana* culture with an intact microbiome. *Ng*^*MB*−^ = *N. gaditana* culture with an attenuated microbiome attendant to being passaged in the presence of antibiotics. *Ng*^*MBTP*^ = The microbiome from *Ng*^*MB*+^ was transplanted into *Ng*^*MB*−^ using FACS. **(A)** Growth profile obtained from *N. gaditana* cell counts using flow cytometry. **(B)** Profile of *B. safensis* titer over time. *B. safensis* titers were obtained by serial dilution of samples from each co-culture flask. The values plotted represent the average and standard deviation, represented by error bars, of three independent cultures. **(C)** Relative OTU (operational taxonomic unit) abundance for bacterial 16S rRNA sequences was sampled on the indicated days. Three distinct *N. gaditana* (Ng) cultures were infected with Bs at the indicated MOI. Asterisks within this panel indicate when the eight core bacteriome members listed in the figure key do not solely compose the entirety of the OTU data for a sample on a particular day.

The 16S rRNA sequencing data showed a steep reduction in *B. safensis* OTUs over time relative to the bacteriome members, even in the absence of Leo2 (Figure 2D, Supplementary Table 1), i.e., by day 3, *B. safensis* was only the sixth most abundant OTU for the *phage-free* cultures inoculated at MOI 0.1 and MOI 1. This suggested that the bacteriome itself may be acting antagonistically to the *B. safensis* infection. To address this, we compared the susceptibility of *N. gaditana* cultures with an attenuated bacteriome to the control (bacteriome-intact) culture. The attenuation was done by passaging *N. gaditana* once on antibiotics (see Methods section) one year prior to these experiments. Additionally, we used fluorescence-activated cell sorting (FACS) to move the bacteriome from the intact culture and restore it to the attenuated culture. We have denoted the three cultures as *Ng*^*MB*+^ (microbiome intact), *Ng*^*MB*−^ (attenuated), and *Ng*^*MBTP*^ (transplant) (Supplementary Figure 2). The antibiotic treatment produced a near-axenic culture and removed most of the bacteriome, whereas the transplant procedure successfully restored the bacteriome (Figure 3C, Supplementary Figure 3, Supplementary Table 2). The three cultures were challenged with *B. safensis* at an MOI of 0.0001, selected based on data from Figure 1. Both the transplant and the control culture survived the low-MOI infection (Figure 3A), and as expected, the bacteriome-attenuated culture crashed in the presence of *B. safensis*. Notably, the titer of *B. safensis* in these cultures over the duration of the experiment was dramatically different, depending on the presence of a bacteriome (Figure 3B). The transplant and control cultures showed a 4–5 log reduction in the *B. safensis* titer after day 1, whereas the titer of *B. safensis* in the *Ng*^*MB*−^ culture was persistently high, at 1 × 10^8^ CFU/ml or greater.

### Phage rescue depends on the presence of the algal bacteriome

**Figure 4:**
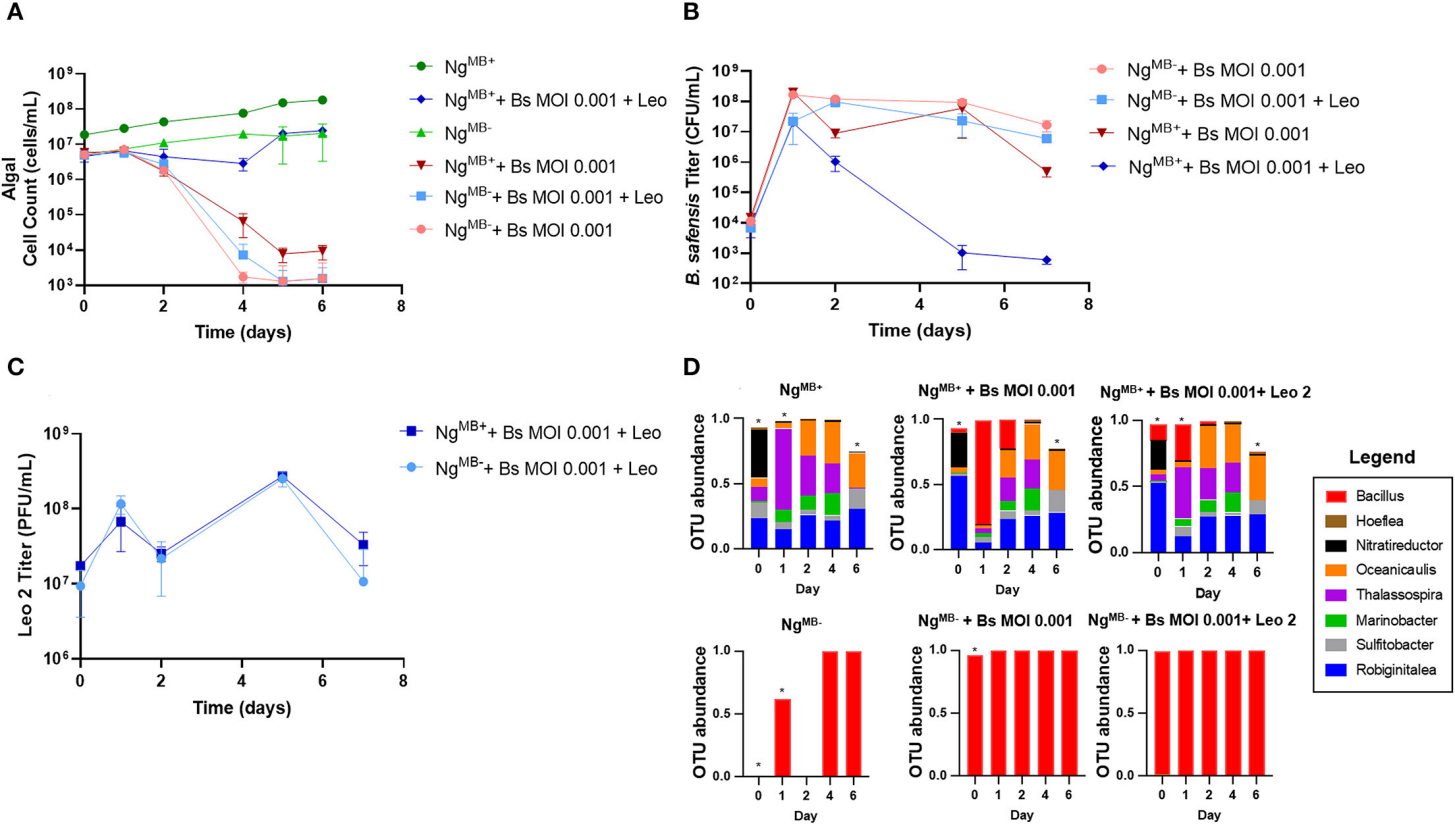
Phage treatment fails to rescue cultures lacking a microbiome. Cultures containing *N. gaditana* (Ng) and/or *B. safensis* (Bs) and/or Leo2 (Leo) were co-cultured at *time* = 0 at the indicated MOIs (ratio of bacteria: algae) for Bs. For phage-seeded cultures, the starting concentration was ~5 × 10^8^ PFU/ml. *Ng*^*MB*+^ = a control *N. gaditana* culture with an intact microbiome. *Ng*^*MB*−^ = *N. gaditana* culture with an attenuated microbiome attendant to being passaged in the presence of antibiotics. **(A)** Growth profile obtained from *N. gaditana* cell counts using flow cytometry. **(B)** Profile of *B. safensis* titer over time. *B. safensis* titers were obtained by serial dilution of samples from each co-culture flask. **(C)** The titer of free phages in cultures containing Ng and/or Bs. The values plotted represent the average and standard deviation, represented by error bars of three independent cultures. **(D)** Relative OTU (operational taxonomic unit) abundance for bacterial 16S rRNA sequences. Asterisks within this panel indicate when the eight core bacteriome members listed in the figure key do not solely compose the entirety of the OTU data for a sample on a particular day.

Given the importance of algal bacteriomes shown here and elsewhere (Kazamia et al., 2012; Liu et al., 2020; Schambach et al., 2020; Lin et al., 2022), it was necessary to determine if phage treatment would be as effective in the bacteriome-attenuated culture. To test this, we infected *Ng*^*MB*+^ and *Ng*^*MB*−^ cultures with *B. safensis* at an MOI of 0.001 with and without phage. As expected, *Ng*^*MB*+^ and *Ng*^*MB*−^ crashed after exposure to *B. safensis*, whereas the uninfected (control) cultures exhibited normal growth (Figure 4A). Surprisingly, phage supplementation rescued the *Ng*^*MB*+^ culture but failed to rescue *Ng*^*MB*−^. The titer of *B. safensis* for *Ng*^*MB*−^ was comparable to phage-untreated cultures that were infected with *B. safensis*. In contrast, the *Ng*^*MB*+^ showed a 4-5 log reduction by day 5 (Figure 4B). Despite the differences in *B. safensis* titer and microalgae growth, the titer of free phage in *Ng*^*MB*+^ and *Ng*^*MB*−^ cultures was comparable (Figure 4C). As observed before, the OTU profile for *Ng*^*MB*+^ (control) cultures was comparable to that of phage-rescued *Ng*^*MB*+^ cultures (Figure 4D, Supplementary Figure 4, Supplementary Table 3). *B. safensis* colonies that were isolated from *Ng*^*MB*−^ cultures were resistant to Leo2 infection (data not shown).

## Discussion

### A mixotrophic model for bacterial pathogenicity

Many outdoor pond production systems assume photoautotrophic growth but are exposed to weather and ecological factors that turn ponds into mixotrophic systems with fluctuating nutrient inputs. We surmise that mixotrophic conditions are more common than previously thought and provide opportunities for invading microbes, contributing to biomass production inconsistencies. There is a recent movement to enhance the feasibility of algal production systems using wastewater (Chinnasamy et al., 2010), or water that contains waste/byproduct carbon sources (Schambach et al., 2020), for microalgae like *Nannochloropsis* that can simultaneously utilize inorganic (CO_2_) and organic carbon substrates (mixotrophy) (Bhatnagar et al., 2011; Villanova et al., 2022). However, the increased availability of organic carbon during mixotrophic growth would be expected to provide an opportunity for pathogenic bacteria invasion. A recent study identified an algae pathogen derived from a poorly performing industrial bioreactor (Fulbright et al., 2016). One of the isolates, *B. pumilus*, exhibited pathogenicity when co-cultured with *Nannochloropsis* sp. in organic carbon-supplemented media but not artificial seawater (Fulbright et al., 2016). Likewise, in our study, we did not observe pathogenicity in photoautotrophic conditions (data not shown). These findings suggest that the availability of organic carbon at the time of infection may determine the level of pathogenicity for a given agent. In other words, microalgae cultures grown photoautotrophically might not be susceptible to pathogen infection until dissolved organic carbon reaches a sufficient concentration, which likely occurs during prolonged growth (Mitra et al., 2015). This question is currently under investigation by our lab. For the current study, the use of mixotrophic conditions functions to remove a possible confounding factor of varying carbon availability at the time of pathogen infection.

### Biotic solutions for biotic problems

As discussed above, outdoor pond cultivation exposes microalgae to highly destructive pathogens. In this study, we discovered that *B. safensis* is a highly virulent pathogen of *N. gaditana* capable of inhibiting cultures at a much lower dose than *B. pumilus* (Fulbright et al., 2016). The difference in virulence might suggest that the two species carry different modes of pathogenicity. It is not known how either species inhibits algal cultures. If *B. safensis* employs a similar pathogenic factor, then a simple explanation for the difference in virulence could stem from differences in the potency or concentration of the factor.

Our data show that neither *B. safensis* infection, phage addition to *B. safensis*-free cultures (control), nor phage rescue (treatment of *B. safensis*-infected cultures) result in extreme shifts in the balance of the bacteriome over time. This indicates that phage Leo2 can rescue pathogen-infected cultures without disrupting the algal bacteriome. Furthermore, the causative agent of *B. safensi*s pathogenicity likely acts on *N. gaditana* itself rather than inhibiting *N. gaditana* by altering the bacteriome. The latter point is reinforced by data showing that the causative agent of *B. pumilus*-mediated inhibition of *N. gaditana* is the secretion of an active molecule (Fulbright et al., 2016).

In other studies, *Bacillus* spp. showed lytic activity against multiple microalgae, including *Chlorella* and *Scenedesmus* (Sheehan et al., 1998; Mars Brisbin et al., 2022), and a *B. cereus* toxin caused the lysis of freshwater microalgae (McBride et al., 2014). Together, these data demonstrate the inhibition of more than one type of alga by multiple species of *Bacillus*, which are ubiquitous soil bacteria. Future studies should investigate the factor responsible for *B. safensis* pathogenicity to *N. gaditana* cultures and whether it is a general feature of *Bacillus* species. It will be important to know if the pathogenicity extends to other microalgae. If follow-on studies show that *Bacillus* species may be a common cause of microalgae crop loss, this will have far-reaching impacts for open pond cultivators since we are not aware of efforts to insulate ponds from soil microbiome contamination.

The bacteriome drives the efficient growth of microalgae (Liu et al., 2020; Schambach et al., 2020), precluding their use to treat pathogen-infected cultures. This problem motivated us to explore the use of phages in microalgae systems. Herein, we showed that phage Leo2 rescued *N. gaditana* cultures from *B. safensis* infection and that the bacteriome was not altered by phage treatment.

### A new countermeasure: phage treatment in microalgae systems

Phages have been previously used to treat bacterial infections of a coral photosymbiont alga (Cohen et al., 2013) and diatoms (Nair et al., 2013). However, to the best of our knowledge, this is the first application of phage to a microalgal production system. Although this study proves the concept at laboratory scales, future studies should examine the feasibility of using phage for commercial crop protection. Future studies should: (1) determine the frequency of crashes caused by bacterial pathogens; (2) identify the causative pathogen(s); (3) develop a cocktail of phages for each pathogen; and (4) compare “phage rescue” to prophylactic use. For the rescue scenario, phage treatments would need to be complemented by a point-of-need diagnostics, informing the selection of phages required for treatment. Conversely, pond stability might be improved with a prophylactic cocktail of phages directed against the most common pathogens. The feasibility of this would depend on the number of pathogens and whether protection can be achieved with minimal doses of phage.

### The native countermeasure: bacteriome's protective role

While the growth-promoting effect of the algal bacteriome is widely known (Kazamia et al., 2012; Liu et al., 2020; Schambach et al., 2020; Lin et al., 2022), the question of whether the bacteriome protects microalgae from bacterial pathogenesis is underexplored. Notably, bacterial communities isolated from crashed rotifer cultures temporarily protected *N. salina* from rotifer grazing (Fisher et al., 2019). We showed here that *N. gaditana* carrying a bacteriome (*Ng*^*MB*+^) survived a low-dose *B. safensis* infection, whereas cultures lacking an intact bacteriome (*Ng*^*MB*−^) crashed after the same treatment (Figure 3). The protective effect was underscored when we re-introduced the microbiome to the *Ng*^*MB*−^culture, restoring the ability of the culture to survive the pathogen infection. Bacteriome-carrying cultures showed a steep reduction in the *B. safensis* titer starting on day 2 (Figure 3B). A simple explanation for the reduced titer of the pathogen is that cultures with an intact bacteriome compete with *B. safensis* for resources (Verschuere et al., 2000).

It would be interesting to test if the bacteriome-imposed growth promotion itself contributes to protection. In principle, strictly protective or strictly growth-promoting members could be distinguished by reintroducing isolates into an axenic algae background and measuring *N. gaditana's* growth rate with and without a pathogen. However, we found it challenging to maintain a near-axenic (*Ng*^*MB*−^) culture, which tended to spontaneously recruit bacteria, including algal pathogens. This point emphasizes the need to improve our understanding and use of protective microbiomes as a standard tool for algal cultivation.

In addition to a lab-scale proof-of-concept for new crop protection tools, the work presented here introduces a simple model for studying host-microbiome relationships. The complexity, dynamics, and variability of natural microbiomes have led researchers to turn to simplified models to better understand homeostasis and disease-related interactions (Wymore Brand et al., 2015). The data presented here suggest that *N. gaditana* (and likely other microalgal systems) may provide an opportunity for a deeper study of phage and pathogen interactions within a system in which the health of the host depends on the integrity and syntrophic interaction of a simple and stable microbiome.

## Data availability statement

The datasets presented in this study can be found in online repositories. The names of the repository/repositories and accession number(s) can be found in the article/Supplementary material.

## Author contributions

BH: Investigation, Formal analysis, Visualization, Writing—review and editing. MM: Investigation, Formal analysis, Visualization, Writing—original draft. ML: Investigation, Data curation, Formal analysis, Visualization. JS: Investigation, Formal analysis, Writing—review and editing. GL: Investigation, Formal analysis. SK: Investigation, Formal analysis, Writing—review and editing. HB: Investigation, Formal analysis. KW: Investigation, Formal analysis, Writing—review and editing. BR: Resources, Investigation. CS: Conceptualization, Funding acquisition, Methodology, Project administration, Resources, Supervision, Validation, Visualization, Writing—original draft, Writing—review and editing. JC: Conceptualization, Funding acquisition, Methodology, Project administration, Resources, Supervision, Validation, Visualization, Writing—original draft, Writing—review and editing.
